# Supplementary material for: Emotional expression through musical cues: A comparison of production and perception approaches
Source: PLoS One. 2022 Dec 30;17(12):e0279605. doi: 10.1371/journal.pone.0279605 (PMC9803112; doi:10.1371/journal.pone.0279605)
Supplement: S1 File — (DOCX) [file pone.0279605.s001.docx]

### Pilot Experiment: Emotional Expressivity of Different Instruments

To determine which instruments had a substantial emotional range and were capable of expressing different emotions, we carried out a pilot study which tested the ability of 27 instruments detailed in S1 Table, to express the following emotions: sadness, joy, calmness, anger, fear, power, and surprise. The following sequence C3-E3-G3-E3-C3 was exported in Logic Pro X using instruments available in the Vienna Symphonic Library (VSL) sound library. The sequence was exported both in *legato* and *staccato* for instruments that supported the aforementioned articulation methods, and one time for instruments such as celesta, marimba, and xylophone which have one articulation setting. Twenty-two participants in an online study rated how much of the aforementioned seven emotions the different instruments were capable of expressing in the musical sequence. Based on the ratings, the instruments were then ranked with respect to their emotional expressivity range within the articulation. This was attained by calculating the maximum emotional expressivity rating difference between the two articulation types for each instrument (instruments that have only one articulation setting were thus omitted from the ranking). S1 Table shows the ranking of the instruments in terms of the emotional expressivity range across the articulation, and the mean rating for each instrument across all seven emotions. Four instruments were selected for each instrument family used in the interface (brass, woodwinds, and strings). We tried to choose instruments that ranked high in the emotional expressivity range. The Vienna horn (ranked #3) and euphonium (ranked #6) were selected as part of the brass ensemble. The cello (#2), violin (#4), and viola (#13) were put forward as part of the strings ensemble. The flute (#1), French oboe (#8), clarinet (#9), and bassoon (#14) made up the woodwinds ensemble.

**S1 Table.** **Ratings of emotional expressivity ability of instruments.**

| Ranking | Instrument | Instrument Family | Expressivity Range | | Mean |
| --- | --- | --- | --- | --- | --- |
| 1 | **Flute*** | Woodwinds | | 2.15 | 2.05 |
| 2 | **Cello*** | Strings | | 1.95 | 2.14 |
| 3 | **Vienna Horn*** | Brass | | 1.80 | 1.93 |
| 3 | Heckelphone |  | | 1.80 | 2.01 |
| 4 | Basset |  | | 1.75 | 1.99 |
| 4 | **Violin*** | Strings | | 1.75 | 2.11 |
| 5 | English Horn |  | | 1.65 | 1.90 |
| 6 | Alto Sax |  | | 1.60 | 2.02 |
| 6 | **Euphonium*** | Brass | | 1.60 | 2.02 |
| 7 | Bass |  | | 1.58 | 2.02 |
| 8 | **French Oboe*** | Woodwinds | | 1.50 | 1.92 |
| 9 | **Clarinet*** | Woodwinds | | 1.45 | 1.98 |
| 10 | Soprano Sax |  | | 1.40 | 2.01 |
| 11 | Trumpet |  | | 1.35 | 2.06 |
| 12 | **Bassoon*** | Woodwinds | | 1.05 | 2.02 |
| 13 | **Viola*** | Strings | | 1.00 | 2.03 |
| 14 | Tenor Sax |  | | 0.95 | 2.03 |
| 14 | Flugelhorn |  | | 0.95 | 2.04 |
| 15 | Cornet |  | | 0.85 | 1.96 |
| NA | Celesta |  | | NA | 2.05 |
| NA | Harp |  | | NA | 1.99 |
| NA | Harpsichord |  | | NA | 2.02 |
| NA | Marimba |  | | NA | 1.89 |
| NA | Organ |  | | NA | 2.24 |
| NA | Piano |  | | NA | 2.07 |
| NA | Vibraphone |  | | NA | 2.02 |
| NA | Xylophone |  | | NA | 1.82 |

Instruments marked in bold and with an asterisk (*****) were taken forward to the study.
