# Supplementary material for: Emotional expression through musical cues: A comparison of production and perception approaches
Source: PLoS One. 2022 Dec 30;17(12):e0279605. doi: 10.1371/journal.pone.0279605 (PMC9803112; doi:10.1371/journal.pone.0279605)
Supplement: S2 File — (DOCX) [file pone.0279605.s002.docx]

### Experiment 1 COVID-19 Safety Measures

Experiment 1 was structured in two parts. The first part of the study required participants to answer a number of demographic questions such as age, gender, and musical expertise. This was administered online via a short survey on Qualtrics to minimise participant and researcher interaction due to COVID-19. Instructions and a video demonstration for the second part of the study (the musical task using *EmoteControl*) were also presented to the participants online. The musical task was done in person. Participants could choose where to carry out the experiment from a number of locations, depending on their personal preference and convenience, with each location consisting of a quiet room with a desk on which to put the apparatus. Speakers were utilised rather than headphones, for hygienic and safety purposes due to COVID-19. Speakers were set to the same volume prior to the experiment. A wireless keyboard and mouse were used rather than the laptop’s own keyboard and mousepad, which were easier to wipe down. All apparatus was sanitised between each participant. Both researcher and participant wore face masks and sat at a distance from each other.
